# Review of the Case of Ann Fooks, with Arguments to Prove Her Having Practised a Deception

**Published:** 1814-03

**Authors:** James Gibbon


*For the Medical and Physical Journal.*

*Review of the Case of Ann Fooks, with Arguments to prove her having practised a Deception; by* Mr. JAMES GIBBON.

IT was my intention to have offered some observations upon the whole of the communication from Dr. Yeats, in your two last Journals, respecting the case of Ann Fooks; but, to my great astonishment, I find such a variation between the published case and a manuscript copy of it, which he favored me with, that I am at a loss how to proceed. More especially have I reason to be surprised at this circumstance, because Dr. Y. has repeatedly observed, that her story never varied, and that he had always found her strictly consistent, although I have reason to think far otherwise. He has assured me, that in repeating her *longest stories* about her *great diversity* of *feelings*, she has scarcely varied a syllable. The Rev. Mr. Grimshaw likewise lays great stress upon the correctness and tenacity of her memory.

When I was called upon to give some reasons why I could not believe the assertions of A. F., I thought it proper to send Dr. Y. a copy of the statement I wrote out. In this statement I did not mention his name. I considered the young woman as being so much under the care of the druggist, as not to make it necessary that Dr. Yeats should be implicated; and should it be satisfactorily proved that he was imposed upon, it would then only have excited a passing smile at the expence of his discernment. He has, however, provoked a controversy, and now accuses me of being the offender.

In consequence of my sending this statement to Dr. Y., he in return sent me his case-book, which, he assured me, contained the *whole* of Ann Fooks's case, to the 5th of May, 1812. The history of her complaints, as there related, agreed so well with the report that she made to Mr. Williams and myself, that I was at the trouble of taking *a full and correct* copy of it. This was done on the 23d of June, 1812.

The variations* may appear trifling to Dr. Y., but to me

---

* In the original history that Dr. Y. took of this case, there was mention made of vomiting fæces; as I think would be evident to any who might be permitted to inspect his case-book.

they they appear far otherwise. In my first paper respecting the case of A. F., I have alluded to this, his original history of her case. Dr. Y. will possibly remember, that he assured me the whole of his notes upon the case of A. F., as taken from her own mouth, at her bed-side, would be found between those leaves of the book which he had doubled down. I copied the whole, which reached to a MUCH LATER PERIOD than that which relates to the additions and alterations that I now refer to. Having sent my statement to Dr. Y. without any injunctions, I feel myself at liberty to ask him, if he can satisfactorily account for these changes, without calling into question the uniform consistency and correctness of A. F.'s memory?

Reserving, therefore, some observations, which I should have thought it incumbent upon me to make, respecting the former part of the case, I will proceed to remark upon the latter part of it.

I readily acknowledge that Dr. Y. evinces much *ingenuity*, and still more plausibility, in the paper before me.

In reporting so long a train of dreadful, and generally incurable symptoms, as concurring in the solitary person of A. F., he must needs have the sympathies of every humane heart. It affords me, therefore, some consolation to be able to assure every person, who may feel interested in the result of the controversy, that this young woman is not more likely to die than Ann Moore of Tutbury was, nor even than Dr. Y., or myself.* This opinion ill accords with the repeated prognostications of Dr. Y.; nevertheless, I will venture to give it in the most unequivocal manner. I am not singular in this opinion.

Upon a hasty reading of his long paper, any person might be induced to suppose that Dr. Girdlestone, Mr. Short, and Mr. Leach, really believe in the tale of urinous vomiting, as related by A. F. I have reason to suppose they utterly disbelieve it. In this paper I shall have occasion to mention the sentiments of Mr. Short upon the subject.

---

* In making this bold declaration, I mean that there is, in my opinion, no symptom of dangerous disease existing in her person, at this time. I deny not but that she may be weakly. Most satisfactorily, however, has it been proved to numbers of persons, that the positive declarations of Dr. Yeats, as to the extent of Ann Fooks's danger, were incorrect. Did she exaggerate, or did Dr. Y. betray a want of medical discernment? The sickly, not the robust, are the most likely to submit to the inconveniences of a feigned and exaggerated illness. But not on this, or any other account, must medical men shut their eyes.

With

With the view of ascertaining that Sarah Triplow, and Lætitia Clarke, were the identical women who positively confessed that they knew nothing about the urinous vomiting, when first questioned by Mr. Short and myself, very soon after the time of its reputed occurrence, I went with Mr. Short this day, Feb. 5, 1814, to their respective houses. We saw them, and ascertained that they were the same persons. Mr. Short and I can testify that they did, upon a second examination, when Dr. Y. was present, most grossly prevaricate. They persist in their self-contradiction.

Let it be clearly understood, A. F. and her mother expressly said to Mr. Short and me, at the time Triplow and Clarke were called to substantiate the assertions of A. F. respecting the urinous vomiting, that they, A. F. and her mother, knew of no other person who had witnessed its occurrence. Hannah Dennis is now brought forward. In addition to the testimony which she was ready to depose before Dr. Yeats, as mentioned in page 118 of the Journal, she told Mr. Short this same day, Feb. 5, in my presence, that she could also depose to having witnessed the movement of the urine, from the swelling of the stomach, to the region of the bladder, by its softening effect, upon the former hardness or rigidity of the lower belly. No doubt, this addition to the evidence which Dr. Y. reports, was made with the view of adding to the importance of the testimony. I ought to apologize for filling your Journal with such trifling matter, but the conduct of Dr. Y. compels me to notice these appendages to his case; and therefore would inform H. Dennis, that Dr. Yeats and I witnessed the existence of this hardness of the lower parts, some weeks after she declared it was effectually removed.

I had long ceased to visit Ann Fooks, but, in consequence of her lodging with S. Triplow, I had an opportunity of seeing her with Mr. Short this morning. Mr. S. questioned her respecting the assertions she formerly made to him about the use of the catheter, and he can testify that she now denies them, yet she is said to have a correct and unerring memory. I could also state upon oath (Dr. Y. being fond of oaths) that A. F. and her mother did this day most grossly (and I suppose wilfully) contradict many of their former assertions made to me, respecting the introduction of the catheter, and Dr. Y.'s observations thereupon.

Mr. Short assures me, that he is as much as ever of opinion that A. F. acted deceptiously in respect to urinous vomiting. I argue for no more.

It is wrong for Dr. Yeats to persevere in misrepresenting the intentions of the parties who signed the "Document."

By

By what construction of language can it be made to mean, that we declared it was impossible for urine ever to be vomited? He ought to recollect, that we only mentioned the impossibility of the occurrence, in the manner, for the period, and with the consequences, that are reported by A. F.

I might challenge Dr. Y. to bring forward one respectable medical character who will undertake to say, that it is possible for a person to exist *ten years* in the dreadful state of suffering which A. F. declares she underwent; and then exhibit such incontestible proofs of a well-nourished, and comparatively healthy body, as she did, at the period of the consultations, which were held soon after the cessation of the repeated urinous vomiting.

I could enlarge very fully upon the subject of altered secretions and vicarious glandular actions; but it is more incumbent upon me to notice the facts of this curious case.

Who would, or ought to believe, in the relation of A. F. and her mother, without demonstrative evidence of its truth? Mr. Williams and I sought for such evidence, with great diligence, but it was not to be found.

I appeal to every candid, judicious, and experienced medical man, whether I ought not to declare the firm belief I entertained of the deception of A. F., when such irrisistible grounds for suspicion presented themselves, as I have reported in my first statement.

Mr. Williams and I were proceeding, step by step, to unfold the mysteries of this case; and only made such slow progress, because A. F. with great adroitness managed to prevent us, by earnest entreaties, from declaring to other medical men our decided opinion, until she thought she could contrive to prevent a glaring detection. More promptness on our part would have settled the question for ever.

Dr. Y. has wandered wide into the field of theory, and has given the public a most marvellous story upon the bare assertion of persons whose testimony ought, in a case like this, to be received with caution, even if there was no ground to suspect deceptious motives. Dr. Y. cannot inform us that the mother of A. F. is a person whose testimony ought to be received, without the greatest suspicions.

I repeat, with certainty, that if A. F. could eat and drink no more than Dr. Y. supposes, there must, for a long time, have been very good cheer for the mother and her neighbours.

Dr. Y. must acknowledge that his own memory is rather incorrect, because he denies that the medical men who signed the document on the 26th of June, 1812, had ever met met before. They all met, and he was of the party, twice during the preceding winter, at the Bedford Infirmary. He perseveres in saying it has an ungracious appearance. I should have been wanting in justice to myself, and in justice to the public, if I had omitted so fair an opportunity of obtaining the sentiments of two of the most respectable medical characters in this part of the kingdom, upon such an extraordinary case. I may say further, in vindication of these gentlemen and myself, that Dr. Yeats is not authorised to make the observations he persists in doing, upon the subject of this medical meeting.

I regret to appear unnecessarily prolix, but as A. F. managed to elude detection by such positive evidence, as was obtained in the case of A. Moore, I am compelled to dwell fully upon the circumstantial evidence which exists against her.

(To be continued.)

