# Corrigenda

**Published:** 1814-04

**Authors:** 


					p. 205, note, for " was mention," read " zua$ no mention,

				

